# Evaluation of Local Pediatric Out-of-Hospital Cardiac Arrest and Emergency Services Response

**DOI:** 10.3389/fped.2022.826294

**Published:** 2022-02-22

**Authors:** Kate McKenzie, Saoirse Cameron, Natalya Odoardi, Katelyn Gray, Michael R. Miller, Janice A. Tijssen

**Affiliations:** ^1^Department of Paediatrics, Schulich School of Medicine and Dentistry, Western University, London, ON, Canada; ^2^Faculty of Medicine, University of Toronto, Toronto, ON, Canada; ^3^Children's Health Research Institute, Lawson Health Research Institute, London, ON, Canada

**Keywords:** pediatric, cardiac arrest, resuscitation, emergency medical services, deviations

## Abstract

**Background:**

Survival after pediatric out-of-hospital cardiac arrest is poor. Paramedic services provide critical interventions that impact survival outcomes. We aimed to describe local pediatric out-of-hospital cardiac arrest (POHCA) events and evaluate the impact of the paramedic service response to POHCA.

**Methods:**

The Canadian Resuscitation Outcomes Consortium and corresponding ambulance call records were used to evaluate deviations from best practice by paramedics for patients aged 1 day to <18 years who had an atraumatic out-of-hospital cardiac arrest between 2012 and 2020 in Middlesex-London County. Deviations were any departure from protocol as defined by Middlesex-London Paramedic Services.

**Results:**

Fifty-one patients were included in this study. All POHCA events had at least one deviation, with a total of 188 deviations for the study cohort. Return of spontaneous circulation (ROSC) was achieved in 35.3% of patients and 5.8% survived to hospital discharge. All survivors developed a new, severe neurological impairment. Medication deviations were most common (*n* = 40, 21.3%) followed by process timing (*n* = 38, 20.2%), vascular access (*n* = 27, 14.4%), and airway (*n* = 27, 14.4%). A delay in vascular access was the most common deviation (*n* = 25, 49.0%). The median (IQR) time to epinephrine administration was 8.6 (5.90–10.95) min from paramedic arrival. Cardiac arrests occurring in public settings had more deviations than private settings (*p* = 0.04). ROSC was higher in events with a deviation in any circulation category (*p* = 0.03).

**Conclusion:**

Patient and arrest characteristics were similar to other POHCA studies. This cohort exhibited high rates of ROSC and bystander cardiopulmonary resuscitation but low survival to hospital discharge. The study was underpowered for its primary outcome of survival. The total deviations scored was low relative to the total number of tasks in a resuscitation. Epinephrine was frequently administered outside of the recommended timeframe, highlighting an important quality improvement opportunity.

## Introduction

Out-of-hospital cardiac arrest is rare in children and is associated with extremely poor outcomes (1, 2). The survival rate of pediatric out-of-hospital cardiac arrest (POHCA) is 10.2% in North America, and survivors often have a new, severe neurological impairment (2). The survival rates following POHCA vary depending on age, etiology, initial rhythm, bystander cardiopulmonary resuscitation (CPR), and other factors (1, 2).

Paramedic services play a critical role in the survival outcomes for POHCA (3–5). However, paramedics' lack of exposure to POHCA due to infrequent occurrence may contribute to a variable response. It is common for paramedics to prioritize extrication and transportation, which have been shown to be associated with worse outcomes (5, 6). Previous research has demonstrated that early on-scene management such as high-quality CPR and the time of medication administration are associated with improved survival (4, 5, 7); as such, resuscitation protocols prioritize these interventions (8). However, Kirves et al. discovered that only 40% of adult patients who experienced out-of-hospital cardiac arrest received care in accordance with the resuscitation recommendations (9). Deviations from hospital protocols for care of pediatric in-hospital cardiac arrests are also associated with worse outcomes (10). In addition, in a POHCA simulation study with non-shockable arrests, many key resuscitation tasks were not routinely performed in line with Pediatric Advanced Life Support guidelines (11). However, deviations from protocols during on-scene management for POHCA events have not been evaluated.

The purpose of this study was to describe the paramedic response to local POHCA events and to assess its impact on patient outcomes. We hypothesized that the paramedic response to POHCA is variable and that deviations from resuscitation protocols are negatively associated with survival.

## Methodology

This was a historical cohort study of POHCA events from an out-of-hospital cardiac arrest registry between January 1, 2012, and June 30, 2020. This study was approved by Western University Research Ethics Board (Project ID: #115304).

### Study Population

This study included children aged 1 day to <18 years old who experienced atraumatic out-of-hospital cardiac arrest in Middlesex-London County, Ontario, Canada. All children who had emergency medical services called to the scene and attempts at chest compressions and/or external defibrillation by Middlesex-London Paramedic Services (MLPS) were included. MLPS services a population of ~450,000 people. Patients were excluded if MLPS did not treat for cardiac arrest, if patients were not transported to hospital following identification of cardiac arrest, or if the primary outcome variable was missing. All patients were divided into age groups: infants (1 day to 12 months), children (1 year to 11 years), and adolescents (12 years to <18 years). The primary outcome for this study was survival to hospital discharge. Secondary outcomes included return of spontaneous circulation (ROSC), survival to hospital admission, neurological status at hospital discharge, and survival to 90 days post-cardiac arrest. Baseline, 6-month, and 12-month Pediatric Cerebral Performance Category (PCPC) post-POHCA scores were collected and reported as per the Pediatric Core Outcome Set for Cardiac Arrest guidelines (12). Events that were excluded were not included in the analysis of the primary or secondary outcomes.

### Data Collection

Ambulance call records (ACR) for all POHCA events in Middlesex-London County from January 1, 2012, to June 30, 2020, were collected using study identification numbers through the Canadian Resuscitation Outcomes Consortium (CanROC) registry, a national de-identified out-of-hospital cardiac arrest registry. The registry includes all documentation required for reporting cardiac arrest for research as per the Utstein criteria (13). The researchers were not blinded to the outcomes of the events during data extraction. There was only one data extractor for the study. The deviation score definitions were objective and agreed on by the study team. The data collected from ACRs and the CanROC registry were combined, along with patient hospital records to create the POHCA database for our study. If there was any discrepancy between CanROC and ACR data, ACR data (as source data) were used.

### Care Deviation Definitions

All MLPS protocols were collated. When local protocols were non-specific, the Heart and Stroke Foundation of Canada's Pediatric Advance Life Support guidelines were used. The protocols guide Primary Care and Advanced Care Paramedics. Primary Care Paramedics can perform CPR and administer limited medications orally or through intramuscular routes. Advanced Care Paramedics have more training which allows them to insert advanced airways, secure IV and IOs, and administer intravascular medications such as epinephrine. Deviations were defined a priori as departure from “best practice” (8, 14–16). We used current protocols to define “best practice” when evaluating deviations across the study period. A deviation was not scored if a Primary Care Paramedic did not administer epinephrine as it is not in their scope of practice. It would be scored a deviation if an Advanced Care Paramedic delayed epinephrine administration as this is within their scope. Adapted from Wolfe et al., we modified eight categories of deviations for the POHCA population: (1) Airway management, (2) Medications, (3) Vascular access, (4) Chest compressions/CPR, (5) Defibrillation, (6) Equipment function, (7) Alerting, and (8) Process timing (10). Each deviation category was subdivided into specific actions for scoring, with the possible data entries of: Yes (deviation occurred), No (deviation did not occur), or Unknown (missing information). An example of a deviation would be delayed epinephrine administration. Epinephrine should be administered directly after IV or IO is secured, and before advanced airway is placed. If epinephrine is administered after another step after the IV is secured or if an advanced airway is placed prior, a deviation was scored. Each patient event was analyzed for deviations. Deviation frequencies were measured as a proportion of the total number of deviations scored. Secondary variables were also defined. “Circulation process of care deviation” (C-DEV) was defined as a deviation from the CPR/chest compression, defibrillation, medication, and vascular access categories (10). Others included “Airway deviation” (A-DEV), defined as any airway deviation that could include delayed advanced airway placement such as multiple attempts, or inappropriate advanced airway placement, and “Process deviation” (P-DEV), defined as any deviation in equipment function, process timing, and alerting categories. There were 83 predefined deviations that could occur, and the number of potential deviations was calculated as all deviations multiplied by the number of events.

### Data Analysis

Descriptive analyses were performed on patient and cardiac arrest characteristics, interventions, deviations, and outcomes; continuous variables were summarized using medians and interquartile ranges, and categorical variables were summarized using frequencies and percentages. Deviations were analyzed as categories and as individual deviations. Bivariate analyses were completed on total deviation categories, composite deviation categories, and demographic characteristics for all outcome variables. A prognostic score for survival likelihood was calculated for each patient using patient and arrest characteristics and known predictors of survival. Each predictor was scored as a +1 or a−1. Positive predictors included adolescent age, witnessed arrest, bystander CPR, shockable rhythm, and drowning as an etiology (17–21). Negative predictors included unwitnessed arrest, no bystander CPR, asystole as an initial rhythm, sudden infant death syndrome as an etiology, baseline PCPC >1, do not attempt resuscitation status, and a severe underlying condition (e.g., Cardiomyopathy, Trisomy 18) (18, 20, 22).

## Results

Between January 1, 2012, and June 30, 2020, there were 52 pediatric patients who experienced an atraumatic POHCA in Middlesex-London County. One patient was excluded as they did not receive CPR by paramedics and were not transported to hospital. Patient and event characteristics are listed in Tables 1, 2, respectively. Patient characteristics were not associated with deviations. ROSC was achieved in 18 (35.3%) patients with 15 (29.4%) surviving to hospital admission. Survival to hospital discharge occurred in 3 (5.8%) patients. Survivors had a new, severe neurological impairment with PCPC scores of 4 at both 6 and 12 months post-POHCA.

**Table 1:** Patient characteristics.

**Characteristics**	**Total (*n* = 51)**
***n* (%)**	***n* (%)**
**Age (years) (median, IQR)**	2 (0 – 14)
Infant (1 day to 12 month)	21 (41.2)
Child (1 year to 11 years)	13 (25.5)
Adolescent (12 years to <18 years)	17 (33.3)
**Male sex**	27 (52.9)
**Pre-existing conditions**	
Cardiac	5 (9.8)
Respiratory	10 (19.6)
Neurological	6 (11.8)
Other	14 (27.4)
**Baseline PCPC category**	
1	40 (78.4)
2	≤ 5
3	≤ 5
4	7 (13.7)
5	≤ 5
Unknown	≤ 5

*Small cells were eliminated for anonymity*.

**Table 2:** Event characteristics.

**Event characteristics**	**Total**
***n* (%)**	**(*n* = 51)**
**Year of event**	
2012–2014	18 (35.3)
2015–2017	12 (23.5)
2018–2020	21 (41.1)
**Bystander witnessed**	16 (31.3)
**Bystander CPR**	40 (78.4)
**Bystander AED used**	≤ 5
**Etiology**	
No obvious cause	32 (62.7)
Drowning	≤ 5
SIDS	≤ 5
Hanging	≤ 5
Other	7 (13.7)
**Initial rhythm**	
Asystole	30 (58.8)
VF/pVT	≤ 5
PEA	13 (25.4)
AED non-shockable	≤ 5
Unknown	≤ 5
**Location**	
Public	7 (13.7)
Non-public/private	44 (86.2)
**Highest level on scene**	
BLS	7 (13.7)
ALS	44 (86.2)
**Time on scene (median, IQR)**	0:13:57 (0:08:06 – 0:23:25)
<10 min	23 (45.1)
10–35 min	28 (54.9)
**Intravenous access**	8 (15.6)
**Intraosseous access**	33 (64.7)
**Airway**	
None	10 (19.6)
Supraglottic	21 (41.2)
ETT	18 (35.2)
Surgical	≤ 5
**Medications**	
Epinephrine	40 (78.4)
Amiodarone	≤ 5
Dopamine	≤ 5
Fluid bolus	13 (25.4)
Other	≤ 5

*Small cells were eliminated for anonymity*.

Deviations were not associated with the primary survival outcome (*p* > 0.05). A total of 188 deviations were scored. We calculated that there were 4,233 potential deviations based on our cohort of 51 patients and the number of potential deviations per unique event. Therefore, 188 deviations represent a small fraction (4.4%) of total possible deviations. All events had at least one deviation, with a median (IQR) of 3 (2–5) deviations per event. Deviations occurred most frequently in the medication category (*n* = 40, 21.3%), but as for individual deviations, a delay to vascular access (*n* = 25, 49%) was the most common. Following medication, the categories with more frequent deviations are process timing (*n* = 38, 20.2%), followed by vascular access (*n* = 27, 14.4%) and airway (*n* = 27, 14.4%), defibrillation (*n* = 26, 13.8%), alerting (*n* = 17, 9.0%), chest compressions/CPR (*n* = 10, 5.3%), and equipment function (*n* = 3, 1.6%). The most common deviations per category are presented in Table 3.

**Table 3:** Number of deviations occuring per event.

**Deviations**	**Event**
***n* (%)**	**(*n* = 51)**
**At least 1 deviation**	**51 (100)**
**C-DEV (CPR, defibrillation, medication, vascular access)**	**39 (76.4)**
**P-DEV (alerting, timing, equipment function)**	**44 (86.3)**
**Airway (total)**	**22 (43.1)**
Intubation attempted; not achieved	4 (7.8)
Bag-mask ventilation; wrong rate	8 (15.7)
No EtCO_2_ monitoring if available for proper airway placement	3 (5.9)
**Medications (total)**	**23 (45.1)**
Incorrect epinephrine dose	4 (7.8)
Original epinephrine administration delay	8 (15.7)
Epinephrine administration interval delay	8 (15.7)
Inappropriate 0.9% NaCl dose	4 (7.8)
0.9% NaCl fluid bolus not given; indicated	6 (11.8)
**Vascular access (total)**	**26 (51.0)**
Delay in obtaining access	25 (49.0)
**Equipment malfunction (total)**	**3 (5.9)**
**CPR/chest compressions (total)**	**10 (19.6)**
Wrong compression to ventilation ratio without advanced airway	7 (13.7)
**Defibrillation (total)**	**22 (43.1)**
Initial monitored rhythm delay	7 (13.7)
Rhythm check; inappropriate intervals	17 (33.3)
**Alerting (total)**	**16 (31.4)**
Base hospital physician patch delay	15 (29.4)
**Timing (total)**	**38 (74.5)**
No extrication/transport after 3rd analysis	14 (27.5)
Time on-scene not optimal	23 (45.1)

**Figure 1:**
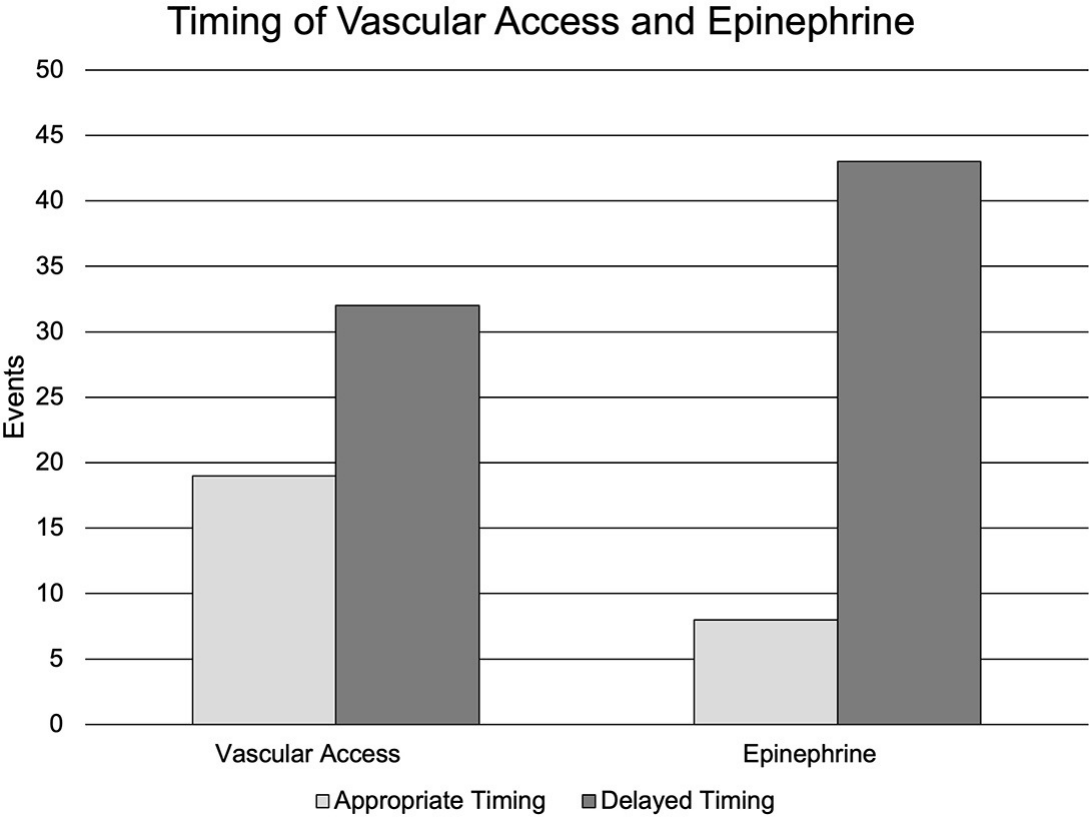
Timing of vascular access and epinephrine. Nineteen (37.2%) events had appropriate vascular access. Thirty-two (62.7%) events had a delay in vascular access. Eight (15.7%) events had appropriately timed epinephrine administration. Forty-three (84.3%) events had a delay in epinephrine administration.

As previously mentioned, delay to vascular access was the most common individual deviation scored (49.0%). The majority of patients had intraosseous (IO) access as the successful method for vascular access (*n* = 33, 64.7%), with an initial attempt success rate of 96.7% compared to the intravenous (IV) initial attempt success rate of 61.5%. The median (IQR) time from MLPS arrival on scene to epinephrine administration was 8.6 min (5.9–10.9). The number of events that had the first dose of epinephrine administered after 5 and 10 min from MLPS arrival on scene was 43 (84.3%) and 15 (29.4%), respectively. MLPS did not administer epinephrine in 11 (21.5%) events. Delay to vascular access and delay to epinephrine administration in each group are reflected in Figure 1. The delay to epinephrine administration could be attributed to a deviation at several decision points during an event. Delayed epinephrine administration caused by a delay in vascular access occurred in 29 (49.0%) events. Delayed epinephrine administration after vascular access was achieved occurred in eight events (15.7%). Primary care paramedics do not administer epinephrine as it does not fall within their scope of practice. Therefore, when only primary care paramedics were on-scene (*n* = 7, 13.7%), a deviation in the sub-category of delay to epinephrine administration was not scored. These events were included in the total number of events with epinephrine administration delay (*n* = 43, 84.3%), regardless of the reason for the delay as described above.

Time on scene (as a continuous variable) was positively associated with total deviations and C-DEV (*p* = 0.01), but there was no significant association with categorical time on-scene. Suboptimal scene time outside of 10–35 min occurred in 24 (47.1%) events. Time on-scene was <10 min in 18 (35.3%) events. POHCA events that occurred in a public setting had significantly more deviations compared to a private setting (*p* = 0.04). ROSC was higher in events with at least one C-DEV (*p* = 0.03).

There were 18 (35.3%) events with Positive Prognostic Scores indicating a higher likelihood of survival based on patient and arrest characteristics prior to MLPS arrival (Table 4). Only 1 (5.5%) of these patients survived to hospital discharge. The number of deviation and the type of deviations in this group were comparable to the whole study cohort; the most common deviations were delay in vascular access (*n* = 9, 50.0%), suboptimal time on scene (*n* = 7, 38.9%), and incorrect rhythm check intervals (*n* = 7, 38.9%).

**Table 4:** Deviations in positive prognostic scored patients.

**Deviation**	**Total**
***n* (%)**	**(*n* = 18)**
Incorrect epinephrine dose	3 (16.7)
Delay of epinephrine administration after vascular access obtained	4 (22.2)
Fluid bolus not given, indicated	3 (16.7)
Delay obtaining vascular access (IV or IO)	9 (50.0%)
Rhythm check at inappropriate intervals	7 (38.9)
Delay of base hospital physician patch	5 (27.8)
Delay in extrication	5 (27.8)
Time on scene not optimal (10–35 min)	7 (38.9)

## Discussion

We described local POHCA events and evaluated paramedic response. Our main findings were: (1) the local POHCA population was similar to other jurisdictions, but the survival rate was lower, (2) deviations from best practice occurred in every event, and (3) we identified important deviations known to be associated with survival, including delay to epinephrine administration and suboptimal time on scene, which are potentially modifiable through quality improvement initiatives.

The patient and arrest characteristics of the local POHCA population in Middlesex-London County are comparable to other studies. The most common initial rhythm was asystole (*n* = 30, 58.8%) and the most common etiology was no obvious cause (*n* = 32, 62.7%), consistent with the current literature (20). The cardiac arrest was witnessed in 31.3% of cases. Our cohort had high rates of bystander CPR (*n* = 40, 78.4%), a factor known to positively influence survival outcomes (2, 5, 23, 24). Our cohort had high rates of ROSC (*n* = 18, 35.3%), double the North American reported average (2), but lower rates of survival to hospital discharge (*n* = 3, 5.8%) and all survivors had new, severe neurological impairments. Our study was underpowered for our primary outcome of survival.

POHCA events are rare, unique, and complex (20). In our cohort, every event had at least one deviation from protocol, with the median (IQR) score of 3 (2–5) deviations per event, highlighting the variability in on-scene management. We considered a deviation rate of 4.4% of all potential deviations to be low. We were not surprised that more deviations occurred when more time was spent on scene. We were intrigued to learn that more deviations occurred for public POHCA events than private (*p* = 0.04). This may be explained by the potential for increased distractions and pressure during a resuscitation where there are more bystanders present.

The common types of deviations that occurred in our study are known to be associated with lower survival (8, 16), and are comparable to those that occurred in a previous simulation study (11). Achieving vascular access through IV or IO is associated with survival in POHCA (5) but this was frequently delayed in our study. Optimal time on scene has been described to be between 10 and 35 min, but this not happen almost half the time, with most scene time deviations of <10 min (5). During pediatric in-hospital cardiac arrest resuscitation, deviating from hospital protocols, particularly care involving medications, CPR, defibrillation, and vascular access, is associated with decreased survival (10). Adherence to protocols during in-hospital cardiac arrest in adults has been shown to increase 30-day survival and lead to more favorable neurological outcomes (25). Though our study was underpowered to show an association between deviations and survival, deviations from protocols are known to influence survival and should be considered as modifiable risk factors to improve survival.

Banerjee argues that time on scene may be less important than what actually happens on scene (7). This theory may also be supported by reports from Japan where scene time is routinely <10 min but survival is 9.8%, with unknown remaining neurological function (26). The majority of our study's events (84.3%) had a delay in epinephrine administration beyond 5 min from MLPS arrival and 29.4% beyond 10 min from MLPS arrival. Our results are better than reported across North America and Taiwan, where, on average, epinephrine was administered at >10 min in 54 and 97%, respectively (4, 24). A recent POHCA simulation study conducted in Oregon found the mean time from paramedic arrival to epinephrine administration was 9.2 min, with 59% of patients receiving either epinephrine after 10 min or no epinephrine at all (11). Our local median time of 8.6 min is less compared to 17.3 min after paramedic arrival in Taiwan and the recent simulation findings (11, 24). Rapid epinephrine administration, namely as soon as possible, is known to increase the chance of survival in pediatric cardiac arrest and it is now reflected in the PALS guidelines (27). For every 1 min delay in epinephrine administration, survival odds decrease by 9% for pediatric cardiac arrest patients and odds decrease 57% when epinephrine is administered after 10 min from paramedic arrival (4).

In our study, almost half of the events (49.0%) had a delay in vascular access. In order to administer epinephrine, vascular access needs to be achieved (20). In the pediatric population, obtaining vascular access can be problematic due to the limited exposure of pediatric resuscitation and challenging anatomy in the poorly perfused patient; thus, this task can be time-consuming, and may distract from high quality CPR. IOs are an alternative route to IVs that are easier and, therefore, quicker to secure but have not been shown to improve cardiac arrest outcomes (28–30). As mentioned previously, IOs had a higher initial success rate (96.7%) in our study compared to IV initial attempts (61.5%). Interestingly, the presence of a caregiver, which is common in POHCA, has been shown to delay the time to IO insertion (31). In our study, bystanders, who were often caregivers, were present for during paramedic resuscitation in 78.4% of events. Therefore, there is a need to investigate the barriers to epinephrine administration and create opportunities for other modes of rapid epinephrine delivery, such as an intramuscular route.

In our study, ROSC was higher in patients who had at least one C-DEV (*p* < 0.05), which we found to be mainly due to vascular access or medication deviations. If the deviation was due to a delay in vascular access or medication administration, this may have translated to reduced interruptions to high quality CPR. Bobrow et al. demonstrated a 4.0% increase in survival to hospital discharge when implementing a minimally interrupted cardiac resuscitation (32). Others have demonstrated that prolonged interruptions to chest compressions are negatively associated with survival (33). Our local ROSC rate is higher than reported in other studies (4, 5, 34), but this is unlikely to be entirely explained by the presumed focus on early high-quality CPR at the expense of vascular access and medication administration.

Although our local ROSC rate was higher than reported studies, the local survival rate was lower compared to Japan and other countries where paramedics do not administer epinephrine (26, 35). A nation-wide study conducted in Japan discovered that spending <5 min on scene for young children and <10 min on scene for older children was associated with higher rates of survival to hospital discharge, regardless of neurological status (26). Because epinephrine is administered 1% of the time on scene in Japan (26), a shorter scene time likely resulted in shorter time to epinephrine administration in the emergency department, which may explain this finding. Another study analyzed North American regional variation in POHCA survival to hospital discharge and discovered that regions with the greatest improvement in survival outcomes had increased bystander CPR, shockable rhythms, and EMS-witnessed cardiac arrest (2). The higher ROSC rate locally is more likely to be attributable to a combination of MLPS interventions such as epinephrine administration, early high-quality CPR, and regular rhythm checks. This group of interventions performed by paramedics may be more important than any individual intervention paired with bystander CPR; however, additional research into post-ROSC factors' impact on survival is required to evaluate the lower local survival rate.

This was a single paramedic agency study with a relatively small convenience sample. A larger sample size is needed to adequately determine whether a link between important deviations and survival exists. There are inherent limitations that occur in retrospective observational studies. Though ACR source data recording was inconsistent at times, the key Utstein variables were collected prospectively through the CanROC Registry in a consistent manner. Data were reviewed from multiple sources, which ensured a higher degree of accuracy and completeness. The definitions used for data collection were object, not subjective, to reduce bias.

## Conclusion

In this retrospective observational study, we discovered that the local POHCA patient and arrest characteristics are comparable to other regions. The MLPS response to POHCA is variable. We reported a high rate of ROSC, but a low rate of survival to hospital discharge. Deviations from MLPS protocols were low relative to the number of potential deviations that can occur in a resuscitation. The study was underpowered for its primary outcome of survival. Although time to epinephrine administration was often delayed, it was better than other reported regions. Future studies should explore the modifiable barriers to rapid epinephrine administration, the optimization of scene time, and the local in-hospital management of POHCA to further investigate opportunities to improve health outcomes.

## Data Availability Statement

The raw data supporting the conclusions of this article will be made available by the authors, without undue reservation.

## Author Contributions

KM, SC, NO, and JT contributed to the design of the study. SC and NO organized the database. KM and KG contributed to data collection. MM performed statistical analysis. KM wrote the first draft of the manuscript. All authors contributed to the article and approved the submitted version.

## Funding

This project was partially funded by the Schulich School of Medicine Summer Research Training Program.

## Conflict of Interest

The authors declare that the research was conducted in the absence of any commercial or financial relationships that could be construed as a potential conflict of interest.

## Publisher's Note

All claims expressed in this article are solely those of the authors and do not necessarily represent those of their affiliated organizations, or those of the publisher, the editors and the reviewers. Any product that may be evaluated in this article, or claim that may be made by its manufacturer, is not guaranteed or endorsed by the publisher.
